# Goal-directed fluid management based on stroke volume variation and stroke volume optimization during high-risk surgery: a pilot multicentre randomized controlled trial

**DOI:** 10.1186/cc9456

**Published:** 2011-03-11

**Authors:** TW Scheeren, C Wiesenack, H Gerlach, G Marx

**Affiliations:** 1 University Medical Center Groningen, the Netherlands; 2 Marienhospital, Gelsenkirchen, Germany; 3 Vivantes - Klinikum Neukoelln, Berlin, Germany; 4 University Hospital RWTH, Aachen, Germany

## Introduction

Perioperative hemodynamic optimization has been shown to be useful to improve the postoperative outcome of patients undergoing major surgery. We designed a pilot study in patients undergoing major abdominal, urologic or vascular surgery to investigate the effects of a goal-directed (GD) fluid management based on continuous stroke volume variation (SVV) and stroke volume (SV) monitoring on postoperative outcomes.

## Methods

Fifty-two high-risk-surgical patients (ASA 3 or 4, arterial and central venous catheter in place, postoperative admission in ICU) were randomized either to a control group (Group C, *n *= 26) or to a goal-directed group (Group G, *n *= 26). Patients with cardiac arrhythmia or ventilated with a tidal volume <7 ml/kg were excluded. In Group G, SVV and SV were continuously monitored with the FloTrac™/Vigileo™ system (Edwards Lifesciences, USA) and patients were brought to and maintained on the plateau of the Frank-Starling curve (SVV <10% and SV increase <10% in response to fluid loading). During the ICU stay, organ dysfunction was assessed using the SOFA score and resource utilization using the TISS score. Patients were followed up to 28 days after surgery for infectious, cardiac, respiratory, renal, hematologic and abdominal complications.

## Results

Group G and Group C were comparable for ASA score, comorbidities, type and duration of surgery (275 vs. 280 minutes), heart rate, MAP and CVP at the start of surgery. However, Group G was younger than Group C (68 vs. 73 years, *P *< 0.05). During surgery, Group G received more colloids than Group C (1,589 vs. 927 ml, *P *< 0.05) and SVV decreased in Group G (from 9.0 to 8.0%, *P *< 0.05) but not in Group C. The number of postoperative wound infections was lower in Group G (0 vs. 7, *P *< 0.01). Although not statistically significant, the proportion of patients with at least one complication (46 vs. 62%), the number of postoperative complications per patient (0.65 vs. 1.40), the maximum ICU SOFA score (5.9 vs. 7.2), and the cumulative ICU TISS score (69 vs. 83) were also lower in Group G. ICU and hospital length of stay were similar in both groups.

## Conclusions

Although the two groups were not perfectly matched, this pilot shows that fluid management based on SVV and SV optimization decreases wound infections. It also suggests that such a GD strategy may decrease postoperative organ dysfunction and resource utilization. However, this remains to be confirmed by a larger study.

